# Metacognitive Monitoring Predicts and Enhances Emotion Management Knowledge

**DOI:** 10.1007/s42761-026-00376-7

**Published:** 2026-06-03

**Authors:** Riley Leckie, Carolyn MacCann, Kit S. Double

**Affiliations:** School of Psychology, The University of Sydney, Sydney, Australia

**Keywords:** Metacognition, Emotional intelligence, Emotion management, Confidence ratings, Self-regulation

## Abstract

Although metacognitive monitoring is widely studied in cognitive domains, its role in emotional functioning remains underexplored. The present research investigates whether traditional measures of metacognitive monitoring, assessed through confidence ratings in cognitive tasks, are associated with and can influence emotional intelligence. In Study 1 (*N* = 226), participants completed a perceptual discrimination task with confidence ratings and a knowledge-based emotion management measure (the STEM-B). Results showed that metacognitive sensitivity (the correspondence between confidence and accuracy) was positively related to emotion management performance, while metacognitive bias (overall confidence) was negatively related. In Study 2 (*N* = 200), participants were randomly assigned to complete the emotion management task either with or without confidence ratings. Participants prompted to make metacognitive judgments performed significantly better, providing causal evidence that metacognitive engagement can enhance emotion management knowledge. These findings suggest that metacognitive monitoring, typically studied in ‘cold’ cognitive contexts, may play a functional role in the regulation of emotion. They also point to new opportunities for improving emotional intelligence through scalable, cognitively grounded interventions.

Metacognition refers to your knowledge and regulation of your cognition (Nelson, 1990; Veenman et al., 2006). Metacognition is typically measured by eliciting online ratings of subjective confidence during cognitive tasks (Fleming, 2024). Monitoring is essential for regulating thought, as it enables individuals to recognize both their current knowledge and gaps in their understanding (Flavell, 1979). This process is critical for performing key cognitive processes, including regulating learning, allocating cognitive resources, and selecting study strategies (Efklides, 2011).

Monitoring also plays an important role in domains that extend beyond traditionally ‘cognitive’ tasks such as perception and reasoning. Notably, monitoring may play a foundational role in emotional intelligence, such as accurately perceiving and understanding your own emotional states (Martinez-Pons, 2000). Although emotions are themselves partly cognitive in nature, the emotion management tasks studied in the emotional intelligence literature differ structurally from the perceptual and reasoning tasks typically used to measure metacognition. Monitoring enables people to recognize shifts in emotion, evaluate their intensity and causes, and determine when regulation is needed. In fact, monitoring is theorized to be a core process in effectively regulating emotions (Sheppes et al., 2015), and should therefore relate to the keystone component of emotional intelligence—emotion management (Mayer et al., 2016).

However, empirical research has not yet examined how objective measures of metacognitive monitoring relate to emotion management. The present studies investigate whether traditionally measured metacognitive monitoring is associated with, and can influence, emotion management.

## Metacognition and Emotion Management

Emotional intelligence, as defined by ability models, comprises four interrelated abilities: perceiving emotions; using emotions to facilitate thought; understanding emotions; and managing emotions (Mayer et al., 2016). Central to these abilities is the capacity to track emotional cues, evaluate one’s internal states, and regulate emotions accordingly. Effective emotion management, in particular, requires individuals to monitor how they are feeling, assess the effectiveness of different emotion management strategies, and adjust their responses over time (Mayer et al., 2016). In this way, emotional intelligence depends not only on emotional sensitivity and knowledge, but also on self-reflective processes akin to metacognitive monitoring.

It is worth noting that metacognitive processes have been studied across a wide range of psychological domains beyond the cognitive tasks on which they are most typically measured. In motivational psychology, metacognitive monitoring has been linked to goal pursuit, academic self-regulation, and adaptive help-seeking (Norman et al., 2019). In clinical psychology, dysfunctional metacognitive beliefs and monitoring processes have been identified as transdiagnostic factors in anxiety, depression, and rumination (Norman et al., 2019). In personality psychology, individuals differ reliably in their tendency to monitor and reflect on their own mental states, and these differences show meaningful associations across different domains (Bürgler & Frey, 2025). The present research adds to this landscape by examining metacognitive monitoring in the domain of emotional intelligence, and suggests that the broad relevance of monitoring processes may extend to emotional functioning.

Although often discussed in parallel, research explicitly linking metacognitive monitoring and emotional intelligence remains limited. Both cognitive and emotional tasks, whether solving a problem or managing a social conflict, demand the ability to monitor performance, recognize when change is needed, and adapt their cognitive strategies accordingly. This shared demand suggests that individual differences in metacognitive monitoring may support emotion management abilities. Indeed, theoretical accounts propose that monitoring may play a foundational role in emotion management flexibility (Briñol et al., 2006; D’Amico & Geraci, 2023). However, empirical evidence for this link is sparse.

Some studies have indirectly examined this connection by exploring how emotional intelligence relates to flexibility in emotion management (Double et al., 2022) or decisions to persist versus switch regulation strategies in response to changing emotional contexts (Ilan et al., 2019). Yet, these studies do not directly assess metacognitive monitoring. Research exploring the relationship between emotional intelligence and metacognition has typically relied on self-report measures (e.g., Albani et al., 2023; Iacolino et al., 2023), which have notable limitations. Self-reported metacognitive ability often shows weak or even negative correlations with objective measures based on confidence ratings in cognitive tasks (Double, 2025). Similarly, self-report measures of emotional intelligence primarily reflect trait-like dispositions and personality traits rather than the ability to manage emotions in real time (Petrides, 2011).

Recent efforts have begun to examine metacognitive monitoring in the context of emotion perception, such as confidence ratings of performance when identifying emotions in voices or facial expressions (Laukka et al., 2021; Lausen & Hammerschmidt, 2020; Sinvani & Fogel-Grinvald, 2024). While these studies suggest that better metacognitive insight is linked to better performance in perceiving emotions, these tasks target only emotion perception abilities, not the more complex emotion management abilities.

The proposed mechanism linking metacognitive monitoring to emotion management knowledge operates through strategic monitoring and control during decision-making about emotional situations. When evaluating which response would be most effective in an emotionally charged scenario, individuals must identify the relevant emotional state, weigh the likely effectiveness of candidate responses, and select among them. These processes parallel the monitoring-and-control cycle in cognitive tasks, in which individuals track their confidence, detect errors, and adjust strategies (Flavell, 1979). Metacognitive sensitivity, the degree to which confidence tracks actual accuracy. may therefore support emotion management knowledge by enabling individuals to discriminate more from less effective candidate responses and to select accordingly.

## Present Studies

The present studies directly test the relationship between objective measures of metacognitive monitoring and emotional intelligence. Study 1 examines whether metacognitive monitoring (confidence ratings in a perceptual decision task), is associated with higher knowledge-based emotion management performance. To test whether this association is causal (monitoring causes higher emotion management task performance), Study 2 uses an experimental design. We test whether eliciting confidence ratings during an emotional management task increases emotion management test performance (compared to a ‘no confidence ratings’ control group). This design builds on evidence that confidence ratings alter performance by enhancing metacognitive monitoring and control processes (Double & Birney, 2017, 2018, 2019; Double et al., 2025). If metacognitive prompting improves emotion management, this provides causal evidence that monitoring contributes functionally to emotion management knowledge, supporting the broader claim that core metacognitive mechanisms underpin both cognitive and emotional intelligence.

## Study 1

### Method

#### Participants

Ethics approval was provided by The University of Sydney Human Ethics Board and all participants provided informed consent. Participants were recruited online via Prolific. Power analysis based on a small-to-moderate effect size (the smallest effect of practical interest) was performed in G*power (power: 80%, *r*=.2), suggesting a minimum sample of 191. To account for exclusions, we recruited 250 participants. We excluded participants if: the attention check item was failed (*n* = 1); we were unable to calculate valid metrics of metacognition (*n* = 22), perceptual task performance was at or near chance level (*n* = 1). The final sample of 226 (51.3% female) had a mean age of 38.52 years (*SD* = 13.31).

#### Materials and Procedure

Participants completed the following two tasks in a counterbalanced order on their own computers. Tasks were coded in jsPsych.

##### Situational Test of Emotional Management – Brief (STEM-B)

The 18-item STEM-B (Allen et al., 2015) assesses emotion management knowledge by presenting participants with text-based scenarios describing emotionally charged situations involving interpersonal conflict, stress, or emotional challenge. Participants select the most effective response from four options. For example, one item reads: “Andre moves away from the city his friends and family are in. He finds his friends make less effort to keep in contact than he thought they would. What action would be the most effective for Andre?” Response options include: (a) Try to adjust to life in the new city by joining clubs and activities there; (b) He should make the effort to contact them, but also try to meet people in his new city; (c) Let go of his old friends, who have shown themselves to be unreliable; (d) Tell his friends he is disappointed in them for not contacting him. Scoring is based on expert consensus: each response option receives a score proportional to the number of experts who rated it as the most effective choice. Items cover a range of emotion management contexts including interpersonal relationships, social transitions, and emotional challenges. The STEM-B has demonstrated good validity and reliability (Allen et al., 2015).

##### Perceptual Decision-making Task

**Fig. 1:**
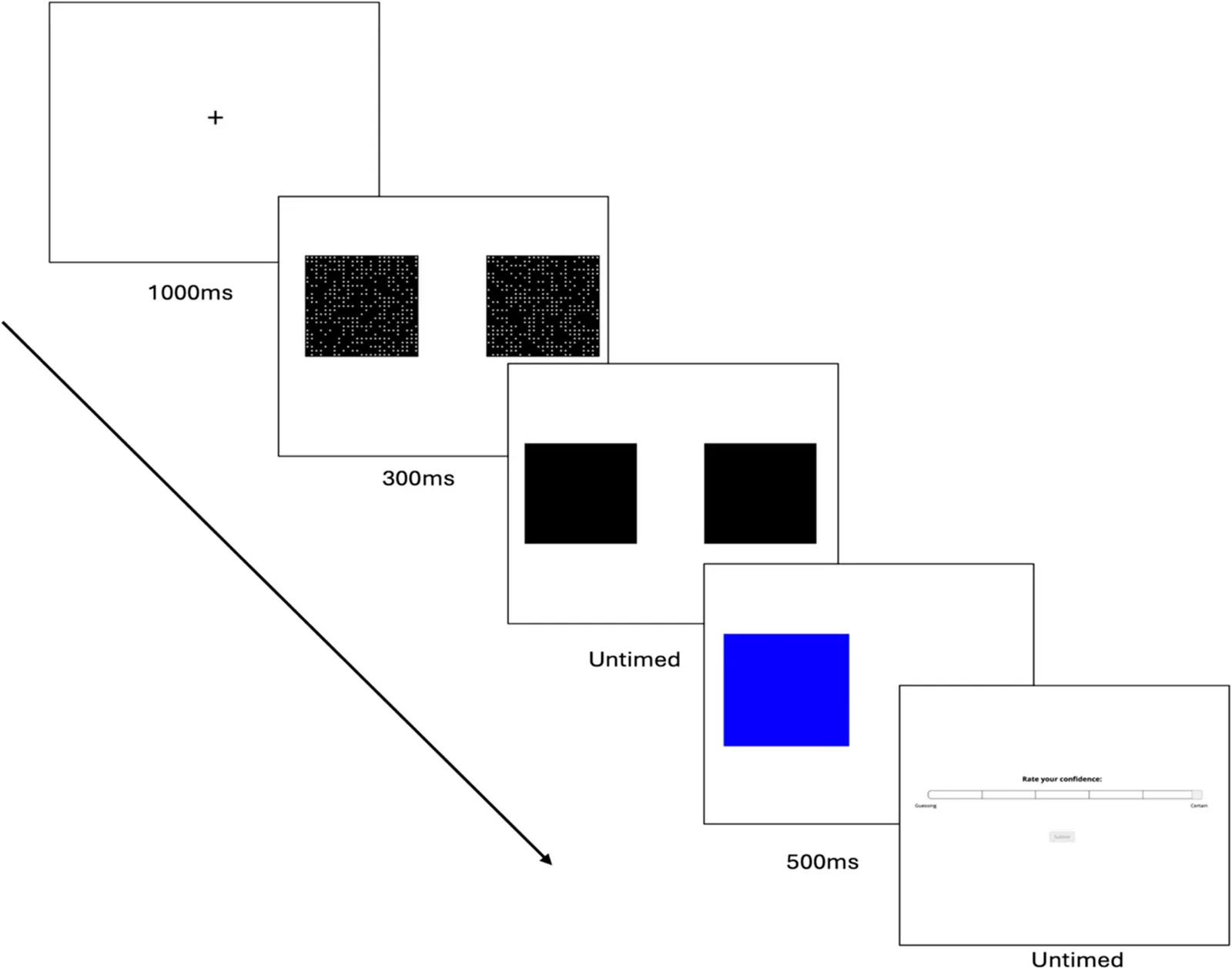
Visual depiction of the perceptual decision-making task used in study 1

This task required participants to judge which of two briefly presented squares contained more dots before making a confidence rating (Double, 2025). As shown in Fig. 1, each trial began with a fixation cross before two black squares were presented on screen for 300ms. Each square contained white dots randomly distributed across 625 potential locations. One square was always half full (313 dots) while the other square contained 1–70 additional white dots. After 300ms, the dots disappeared, and participants indicated whether the left or right box had more dots (using ‘w’ or ‘e’ keys, respectively). Participants had unlimited time to make their decision. After each perceptual decision, participants’ responses were highlighted blue for 500ms before participants rated their confidence on a 6-point scale from “Guessing” to “Certain”.

As signal detection measures of metacognition require a constant level of difficulty (Fleming & Lau, 2014), we used a two-down, one-up staircasing procedure to maintain a constant level of performance (with the dot difference decreasing after two consecutive correct responses and increasing after one consecutive incorrect response) during the experiment and across participants. Equal step sizes for steps up and down were used. Staircasing began in the practice block, with step size calculated in log space, with a starting point of 4.2 (+ 70 dots), changing by ± 0.4 for the first five trials, ± 0.2 for the next five trials and ± 0.1 for the rest of the task.

Using signal-detection theory (Fleming & Lau, 2014), we calculated three measures of metacognition. First, metacognitive bias was operationalized as participants’ mean confidence rating across all trials. Because the staircasing procedure equated accuracy across participants, mean confidence reflects a response bias rather than veridical performance. Second, metacognitive sensitivity (meta-d’) was calculated using the HMeta-d MATLAB toolbox (Fleming, 2017), and reflects how well confidence ratings discriminate between correct and incorrect trials, a higher meta-d’ indicates that confidence more closely tracks accuracy. Third, metacognitive efficiency (meta-d’/d’) indexes metacognitive sensitivity relative to task performance (d’), and allows sensitivity to be compared across participants who may differ in overall accuracy. Perceptual task performance itself was indexed by d’, calculated using standard signal-detection theory; because d’ enters into the calculation of metacognitive efficiency, it is reported alongside the metacognitive measures in Table 1.

### Transparency and Openness

We report how we determined our sample size, all data exclusions, all manipulations, and all measures in the study relevant to the current research questions. All online supplemental materials, data, analysis code, and research materials are available on the Open Science Framework: https://osf.io/5gtkd. Note that the STEM-B items are able in full at a separate OSF link (https://osf.io/mqp2x/). Study 1 was not preregistered. It was designed as an initial exploratory study to examine whether objective metacognitive monitoring measures were associated with emotion management knowledge; this informed the hypotheses and analysis plan for the preregistered Study 2. Consistent with open science best practices, we report all analyses conducted and note Study 1 findings should be interpreted with appropriate caution. We report all relevant studies and analyses that were conducted.

## Results and Discussion

Descriptive statistics and correlations are presented in Table 1. Two of the three metacognition measures were significantly correlated with STEM-B performance: (1) metacognitive sensitivity had a positive effect (when confidence better tracked accuracy on the perceptual task STEM-B performance was higher) and (2) metacognitive bias had a negative effect (higher bias was linked to lower STEM-B performance). Metacognitive efficiency was not significantly related to STEM-B performance. Taken together, our study presents preliminary evidence that metacognition is related to emotional intelligence, albeit weakly.

Intercorrelations among metacognitive measures are also shown in Table 1. Metacognitive sensitivity and efficiency were strongly correlated (*r* =.75, *p* <.001), as expected given that efficiency is calculated as the ratio of sensitivity to task performance (meta-*d*’/*d*’). In contrast, metacognitive bias was not substantially correlated with either sensitivity or efficiency (both rs < 0.10), consistent with evidence that confidence level and confidence accuracy are largely orthogonal dimensions (Fleming, 2017). These patterns suggest that sensitivity and efficiency largely capture a common latent monitoring dimension, whereas bias reflects an independent tendency bias in average confidence.

**Table 1:** Descriptive statistics and correlations

	M (SD)	2	3	4	5
1. STEM-B	10.14 (2.21)	0.11	0.16*	0.08	−0.16*
2. Perceptual performance (d)	0.84 (0.26)		0.33***	−0.26***	−0.02
3. Metacognitive Sensitivity	0.69 (0.39)			0.75***	0.06
4. Metacognitive Efficiency	0.86 (0.57)				0.02
5. Metacognitive Bias	3.86 (0.91)				

* *p* <.05, *** *p* <.001. Perceptual performance (d’) refers to discrimination accuracy on the perceptual decision-making task, calculated using signal detection theory. It is included as the denominator of metacognitive efficiency (meta-d’/d’)

## Study 2

### Method

The study was preregistered (https://aspredicted.org/z5fq-58cx.pdf).

### Participants

Participants were recruited online via Prolific. Power analysis in G*power (power: 80%, *d*=0.40), used a target effect size drawn from reactivity in cognitive tasks (Double et al., 2018). A preregistered sample size of 200 was recommended and collected (*M*_age=_39.66, SD = 14.31; 49.5% female). Participants were randomly allocated to complete the STEM-B either with confidence ratings (*n* = 100, CR condition) or without ratings (*n =* 100, No-CR condition). Consistent with the preregistration, participants who failed an embedded attention check item (which instructed participants to select a specific response) were excluded prior to analysis. No participants failed this check, and the full recruited sample of 200 was retained.

### Materials and Procedure

The STEM-B was administered in the same format as Study 1. The perceptual decision-making task was not included in Study 2; the only manipulation was whether participants made confidence ratings after each STEM-B response (CR condition) or did not (No-CR condition). In the CR condition, confidence ratings were collected after each response. Participants rated their confidence on a 6-point scale from “Guessing” to “Certain”. Participants in the No-CR condition, did not make confidence ratings.

### Results

#### Accuracy

An independent-samples *t*-test indicated that the CR condition (*M* = 10.96, *SD* = 1.95) performed significantly better than the No-CR condition (*M* = 10.33, *SD* = 2.41), t(198) = 2.01, *p*=.046, *d* = 0.28.

#### Response Time

We additionally conducted an exploratory (non-preregistered) analysis of median STEM-B response times to examine whether the confidence rating manipulation influenced deliberation time. Median response time was used in preference to mean response time due to the right-skewed distribution of response times. The No-CR (M = 19,785 ms, SD = 8,731) and CR (M = 20,542 ms, SD = 8,200) conditions did not differ significantly in response time, t(198) = 0.63, *p* =.528, d = − 0.09. This null result suggests that the performance benefit in the CR condition was not simply due to spending more time on each item. This analysis was not preregistered and should be interpreted with appropriate caution.

## Discussion

The present studies provide novel empirical support for the theorized link between metacognitive monitoring and emotional intelligence. Across two studies, we demonstrate that metacognitive monitoring, a core cognitive process typically studied in decision-making and learning contexts, is meaningfully related to, and can influence, emotion management knowledge, a key component of ability-based emotional intelligence.

Study 1 was the first to show that individuals with greater metacognitive monitoring ability perform better on a knowledge-based emotion management performance test. This finding aligns with theoretical models that emphasize the importance of monitoring and self-evaluation for both cognitive and emotional regulation (Briñol et al., 2006). Just as metacognitive monitoring supports cognitive control and strategy adjustment in problem-solving tasks (Birney et al., 2017; Double et al., 2025; Muncer et al., 2022), it may also support the dynamic regulation of emotional responses, for example, by helping individuals recognize when to shift strategies from distraction to reappraisal. While prior research has inferred a connection between metacognition and emotional intelligence based on self-report data (Albani et al., 2023; Iacolino et al., 2023) or confidence ratings in emotion recognition tasks (Laukka et al., 2021; Sinvani & Fogel-Grinvald, 2024), the current findings extend this work by showing that monitoring abilities measured using ‘cold’ cognitive tasks relate to more complex emotional regulation skills. Metacognitive efficiency was not significantly related to emotion management scores. Efficiency (meta-d’/d’) indexes metacognitive sensitivity after statistically controlling for task performance (d’). The null result for efficiency, in contrast to the significant result for raw sensitivity, suggests that the relevant predictor of emotion management is the absolute quality of metacognitive monitoring, rather than monitoring precision relative to task performance. Individuals who are both higher performers on the perceptual task and show greater metacognitive sensitivity may be more attuned to performance differences in the emotion management domain as well, but this relationship is not specific to metacognitive monitoring above and beyond task performance per se. Future research should examine whether this pattern replicates across different cognitive tasks and levels of difficulty.

Study 2 builds on this foundation by testing whether metacognitive monitoring can causally influence emotion management performance. We found that participants who made confidence ratings during a knowledge-based emotion management performance test performed significantly better than those who did not. This reactivity to metacognitive prompting is consistent with findings from cognitive domains, where prompting confidence ratings enhances strategic reflection and performance (Birney et al., 2017; Double & Birney, 2019). Our results suggest that similar mechanisms may operate in emotional contexts: eliciting confidence ratings may encourage participants to reflect more deeply on their responses, attend to relevant emotional cues, or evaluate the appropriateness of regulatory strategies in real time. These findings reinforce the view that metacognitive monitoring plays an active, functional role in supporting self-regulation across both cognitive and emotional domains.

Importantly, the second study also clarifies the interpretation of prior studies that incorporated confidence ratings into emotional tasks without control conditions (e.g. Laukka et al., 2021; Sinvani & Fogel-Grinvald, 2024). The absence of control groups in those studies made it unclear whether observed associations reflected stable individual differences in metacognitive ability or were partially driven by the performance-enhancing effects of metacognitive prompting. Our inclusion of a control condition provides the first evidence that confidence ratings themselves may enhance emotional performance, highlighting the need for future studies to account for such reactivity effects.

Finally, these findings offer a promising direction for emotional intelligence interventions. Many existing programs are time-intensive and lack clear mechanistic targets (Lim & Lau, 2021). In contrast, our results suggest that simple metacognitive prompts, such as eliciting confidence ratings, may offer a scalable, theoretically grounded way to enhance emotion management skills. By targeting the self-monitoring processes thought to underpin emotional intelligence, such interventions may improve outcomes in a more efficient and cognitively principled manner.

Why might metacognitive monitoring support emotion management knowledge as indexed by the STEM-B? Performance on knowledge-based emotional intelligence tasks requires participants to evaluate multiple candidate responses and identify the most effective option. Metacognitive monitoring is well suited to support this process: individuals with greater monitoring sensitivity should be better able to detect uncertainty about which option is most appropriate and to deliberate more carefully when this uncertainty is high. The Study 2 manipulation directly engaged this process, prompting participants to rate their confidence in each response, and produced a corresponding improvement in performance. This is consistent with a mechanistic account in which metacognitive engagement supports the selection of more effective emotion management responses, rather than necessarily reflecting changes in online regulatory ability.

## Limitations and Future Directions

While Study 1 linked metacognitive monitoring on a perceptual task to emotion management, future research could extend these effects to other cognitive tasks (e.g., memory, reasoning) and emotional abilities (e.g., perception, understanding) to establish generalizability. While Study 2 provides causal evidence, the manipulation was minimal (presence/absence of confidence ratings). Future studies could explore whether varying the timing, frequency, or format of metacognitive prompts yields stronger or more sustained effects. Additionally, the mechanisms through which metacognitive prompting improves emotion management remain to be clarified. Effects may be driven by increased self-awareness, strategic reflection, or greater attentional focus. Finally, while studies were adequately powered, larger and more diverse samples could examine potential moderating variables, such as personality traits, stress levels, or baseline emotional intelligence.

A further limitation concerns the nature of the emotion management measure. The STEM-B assesses knowledge of effective emotion management strategies in hypothetical scenario-based format, rather than the online implementation of regulation in response to personally experienced emotion. While scenario-based performance measures are widely used in the emotional intelligence literature and have good validity (Allen et al., 2015; Mayer et al., 2016), future research should examine whether the present findings extend to real-time emotion management paradigms, such as those using laboratory mood inductions or ecological momentary assessment.

The present findings may also have implications for metacognition research in adjacent fields. In clinical psychology, dysfunctional metacognitive monitoring (characterized by ruminative self-focus or inflexible appraisals of one’s internal states) may contribute to difficulties in emotion management. In motivational and educational psychology, the monitoring processes identified here as relevant to emotion management may intersect with self-regulated learning and goal pursuit. Future work should examine whether the metacognitive-emotional intelligence link we report is moderated by individual differences in metacognitive ability/style studied in these adjacent fields.

## Conclusion

Results provide the first evidence that metacognitive monitoring abilities are positively associated with, and can enhance, emotion management knowledge. By bridging cognitive and emotional domains, the findings highlight monitoring as a shared self-regulatory mechanism and suggest new avenues for theoretically grounded interventions to improve emotional functioning.

## Additional Information

### Data availability

All online supplemental materials, data, analysis code, and research materials are available on the Open Science Framework: https://osf.io/5gtkd/?view_only=667005ca014745a2a26fde28369fb468.
